# Supplementary figures and images for: Physiologically relevant aspirin concentrations trigger immunostimulatory cytokine production by human leukocytes
Source: PLoS One. 2021 Aug 24;16(8):e0254606. doi: 10.1371/journal.pone.0254606 (PMC8384208; doi:10.1371/journal.pone.0254606)

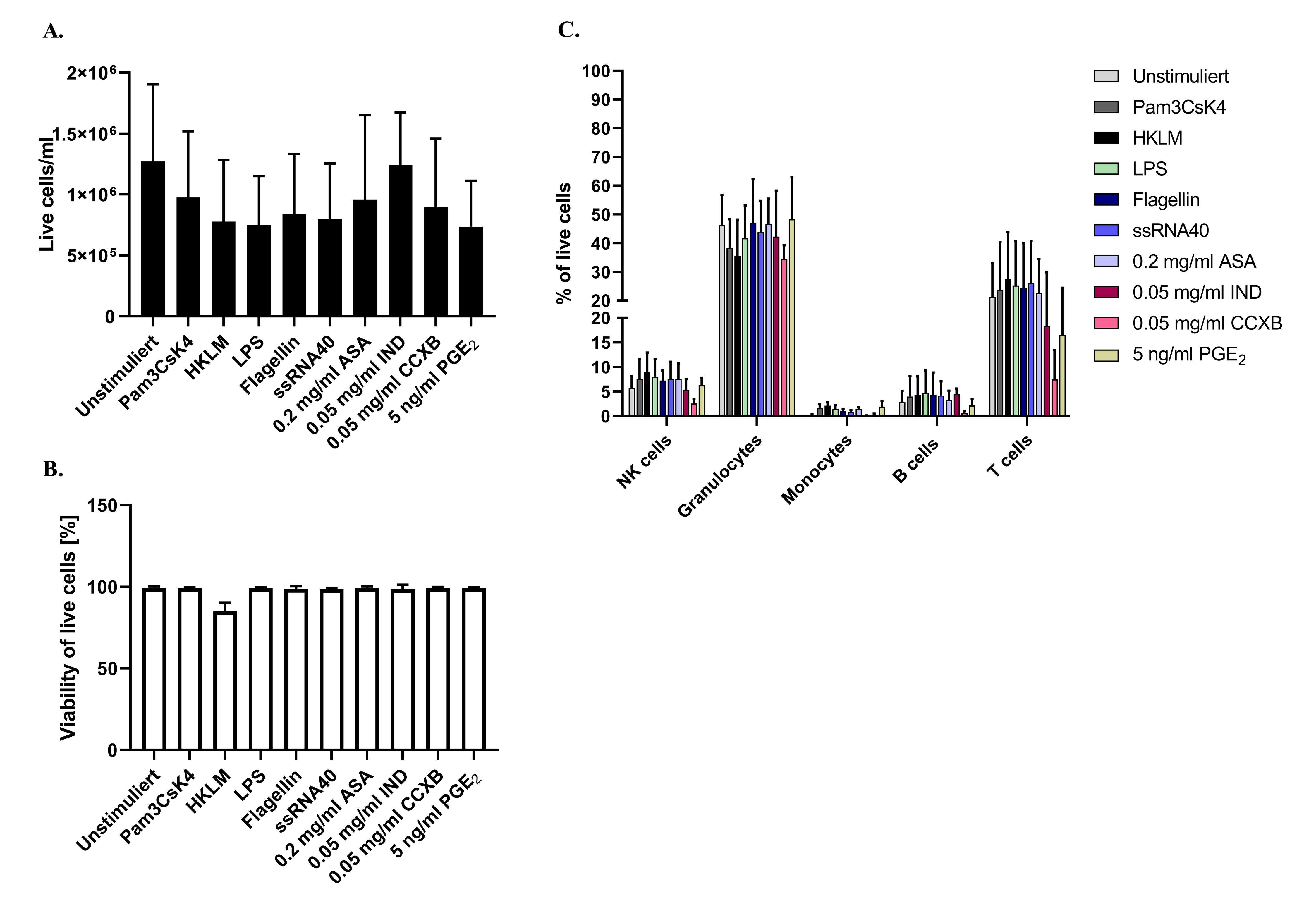

Supplement: S1 Fig — Citrate-anticoagulated blood was treated with TLR ligands, 0.2 mg/ml ASA, 0.5 mg/ml IND, 0.5 mg/ml CCXB, 10 ng/ml PGE2 or vehicle (unstimulated). The absolute number of living cells (A), the viability of cells (B), and the cellular composition within live cells (C) were analyzed by flow cytometry. Data represent three independent experiments, each performed in triplicate. Bars indicate the mean ± SD. (TIF) [file pone.0254606.s001.tif]
